# Investigating the clinical significance of OAS family genes in breast cancer: an in vitro and in silico study

**DOI:** 10.1186/s41065-024-00353-9

**Published:** 2024-12-05

**Authors:** Jinjun Lu, Lu Yang, Xinghai Yang, Bin Chen, Zheqi Liu

**Affiliations:** 1 Department of General Surgery, Nantong Haimen People’s Hospital, NanTong, JiangSu 226100 China; 2 Department of Clinical Laboratory, Chongqing University Cancer Hospital, Chongqing, 400030 China; 3 Department of TCM Gynecology, Hangzhou TCM Hospital Affiliated to Zhejiang Chinese Medical University, Hangzhou, Zhejiang 310000 China

**Keywords:** OAS genes, Breast cancer, Biomarker, Prognosis, Treatment

## Abstract

**Background:**

Breast cancer is the most common malignancy among women worldwide, characterized by complex molecular and cellular heterogeneity. Despite advances in diagnosis and treatment, there is an urgent need to identify reliable biomarkers and therapeutic targets to improve early detection and personalized therapy. The OAS (2′-5′-oligoadenylate synthetase) family genes, known for their roles in antiviral immunity, have emerged as potential regulators in cancer biology. This study aimed to explore the diagnostic and functional relevance of OAS family genes in breast cancer.

**Methodology:**

Breast cancer cell lines and controls were cultured under specific conditions, and DNA and RNA were extracted for downstream analyses. RT-qPCR, bisulfite sequencing, and Western blotting were employed to assess gene expression, promoter methylation, and knockdown efficiency of OAS family genes. Functional assays, including CCK-8, colony formation, and wound healing, evaluated cellular behaviors, while bioinformatics tools (UALCAN, GEPIA, HPA, OncoDB, cBioPortal, and others) validated findings and explored correlations with clinical data.

**Results:**

The OAS family genes (OAS1, OAS2, OAS3, and OASL) were found to be significantly upregulated in breast cancer cell lines and tissues compared to normal controls. This overexpression was strongly associated with reduced promoter methylation. Receiver operating characteristic (ROC) analysis demonstrated high diagnostic accuracy, with area under the curve (AUC) values exceeding 0.93 for all four genes. Increased OAS expression correlated with advanced cancer stages and poor overall survival in breast cancer patients. Functional analysis revealed their involvement in critical biological processes, including immune modulation and oncogenic pathways. Silencing OAS genes in breast cancer cells significantly inhibited cell proliferation and colony formation, while unexpectedly enhancing migratory capacity. Additionally, correlations with immune cell infiltration, molecular subtypes, and drug sensitivity highlighted their potential roles in the tumor microenvironment and therapeutic response.

**Conclusion:**

The findings of this study established OAS family genes as potential biomarkers and key players in breast cancer progression, offering promise as diagnostic biomarkers and therapeutic targets to address unmet clinical needs.

## Introduction

Breast cancer, a pervasive malignancy affecting both men and women, arises from the uncontrolled growth of cells within the breast tissue [1–3]. It is a complex and heterogeneous disease with multiple subtypes, each exhibiting distinct molecular and clinical characteristics [4–7]. While the exact causes of breast cancer are multifactorial [8, 9], several risk factors contribute to its development. Genetic predisposition, particularly mutations in the BRCA1 and BRCA2 genes, family history, hormonal factors such as early onset of menstruation or late menopause, and exposure to estrogen-related medications are known risk factors [10].

The majority of breast cancer patients face a survival duration of less than 5 years, and the incidence of breast cancer continues to rise annually [11]. While various therapies exist for treating different breast cancer subtypes, such as established hormonal therapy for HER-2 positive cases, the effectiveness of HER2-based therapies is anticipated to be limited in basal-like breast cancer (BLBC) [12–14]. This is due to BLBC typically exhibiting low or no expression of these oncogenes. Despite expressing some normal myoepithelial cell markers like cytokeratins (CK5/6, CK14, and CK17), HER1, and c-KIT, these markers fail to fully capture the distinctive characteristics of BLBC [12, 13, 15, 16]. Given this situation, there is an urgent need to explore more dependable biomarkers for breast cancer.

The OAS gene family encodes enzymes involved in innate immunity, particularly in response to viral infections [17]. OAS proteins, including OAS1, OAS2, OAS3, and OASL, synthesize antiviral 2’-5’ oligoadenylates, activating RNase L to degrade viral RNA, serving as crucial defenders against viral threats [18, 19]. OAS genes have been associated with diverse conditions such as innate immune-activated diseases [17], HIV infection [20], and chronic skin disease [21]. In the context of cancer, the OAS family has significant roles in tumorigenesis, immune modulation, and response to therapy. In lung cancer, particularly non-small cell lung cancer (NSCLC), OAS gene expression is dysregulated. Elevated levels of OAS proteins may contribute to the inflammatory tumor microenvironment, promoting cancer cell survival and proliferation [22, 23]. Studies have suggested that OAS proteins might serve as biomarkers for lung cancer prognosis [22, 23], as their expression levels correlate with disease stage and patient survival. In hepatocellular carcinoma (HCC), OAS family genes, particularly OAS1 and OAS2, play a role in the tumor immune response [24, 25]. These genes are involved in antiviral defense mechanisms that may affect the progression of liver cancer, especially in patients with chronic hepatitis infections, where OAS gene activation could influence the hepatic immune response and alter the tumor microenvironment [26, 27]. In pancreatic cancer, the expression of OAS genes is altered, influencing cancer-associated inflammation [28]. OAS proteins regulate the tumor microenvironment by modulating immune responses, and the dysregulation of OAS gene expression in pancreatic ductal adenocarcinoma (PDAC) is associated with immune evasion and may affect the efficacy of immunotherapies and the overall prognosis of patients [29, 30]. Similarly, in colorectal cancer, OAS genes, particularly OAS2, have been linked to immune surveillance and tumor progression [25]. The inflammatory response mediated by OAS proteins contributes to the cancer-related inflammation that supports tumor growth and metastasis [31]. OAS2 expression has been associated with better patient prognosis, highlighting the potential of OAS genes as therapeutic targets in colorectal cancer [32]. These genes hold promise as potential biomarkers for early cancer detection, prognosis, and therapeutic intervention. However, the precise role of the OAS family in breast cancer is still unclear, highlighting the need for extensive research.

In this current study, we investigated the diagnostic and prognostic implications and the therapeutic significance of the OAS gene family in breast cancer through a combination of in silico analysis and molecular experiments. Such insights hold the potential to inform future clinical strategies, personalized treatment approaches, and the development of précised therapeutic interventions in the ongoing battle against breast cancer.

## Methodology

### Cell lines

BRCA cell lines HOC1, HOC7, HOC8, and IGROV1 were cultured in DMEM (Invitrogen, Grand Island, NY, USA #11995) with 10% FBS (Hyclone, Logan, Utah, USA #sv30014.03). OAW42 and CAOV3 were maintained in DMEM with 10% FBS, supplemented with sodium pyruvate (Sigma St. Louis, MO, USA, #S8636), 20 ug/mL insulin (Invitrogen #12585-014), l‐glutamine (Sigma #G7513), or NEAA (Sigma #M7145)/glucose, respectively. FUOV1 was cultured in DMEM: F12 (Invitrogen #11330) with 10% FBS. OVCA420, OVCA432, OVCA433 were grown in EMEM (ATTC, Manassas, VA, USA, #30‐2003) with 10% FBS. SW626 was cultured in Leibovitz’s L15 (ATTC #30.2008) with 10% FBS, without CO_2_. IOSE29 and IOSE80 were maintained in M199:MCDB105 (Invitrogen #11150, Sigma #M6395) with 5% FBS. ES2 cells were grown in McCoy’s 5a (Invitrogen #116600) with 10% FBS and l‐glutamine. Control cell lines, including MCF-10 A, MCF-12 A, HMEC, 184B5, and 76 N-TERT, were cultured in DMEM: F12 (Invitrogen #11330) with 10% FBS.

### DNA extraction

The extraction of DNA was conducted following the manufacturer’s instructions, utilizing the Qiagen QIAamp DNA Mini Kit (Cat # 51304). Briefly, cell or tissue samples were lysed using the provided lysis buffer, and proteins and other contaminants were removed through a series of washes using the kit’s proprietary buffers. The extracted DNA was subsequently quantified using a NanoDrop spectrophotometer (Thermo Fisher Scientific, Cat # 2000) and stored at -20 °C for further analysis.

### RNA extraction

RNA extraction was carried out using the Qiagen RNeasy Mini Kit (Cat # 74104), following the manufacturer’s instructions. Briefly, the tissue or cell samples were first lysed with the provided lysis buffer (RLT buffer), which ensures efficient disruption of cell membranes and denaturation of RNases. After lysing the samples, the lysate was applied to the RNeasy spin column, where total RNA was bound to the silica membrane. he RNA concentration and quality were assessed using a NanoDrop spectrophotometer (Thermo Fisher Scientific, Cat # 2000) by measuring the absorbance at 260 nm.

### Reverse transcription quantitative polymerase chain reaction (RT-qPCR) analysis

The RT-qPCR was performed following the protocol provided with the Bio-Rad iTaq Universal SYBR Green Supermix (Bio-Rad Laboratories, Hercules, CA, USA). Custom-designed primers specific to the target genes were synthesized by Integrated DNA Technologies (IDT, Coralville, IA, USA). To ensure accurate quantification, mRNA expression levels were normalized against the reference gene GAPDH. The relative expression levels of the target genes were calculated using the comparative threshold cycle (Ct) method, employing the ΔΔCt approach. The primer sequences for RT-qPCR were as follows:

GAPDH-F 5’-ACCCACTCCTCCACCTTTGAC-3’.

GAPDH-R 5’-CTGTTGCTGTAGCCAAATTCG-3’.

OAS1-F: 5’-AGTTGACTGGCGGCTATAAAC-3’.

OAS1-R: 5’-GTGCTTGACTAGGCGGATGAG-3’.

OAS2-F: 5’-AGGTGGCTCCTATGGACGG-3’.

OAS2-R: 5’-TTTATCGAGGATGTCACGTTGG-3’.

OAS3-F: 5’- GAAGGAGTTCGTAGAGAAGGCG-3’.

OAS3-R: 5’-CCCTTGACAGTTTTCAGCACC-3’.

OASL-F: 5’-CCCTTGACAGTTTTCAGCACC-3’.

OASL-R: 5’-CTTCAGCTTAGTTGGCCGATG-3’.

### Bisulfite sequencing analysis

#### Library preparation for targeted bisulfite sequencing analysis

Initially, 1 µg of total DNA was sheared into fragments of approximately 200–300 base pairs using the Bioruptor Pico Sonication System (Diagenode, Denville, NJ, USA). The resulting DNA fragments were then subjected to a series of enzymatic treatments to repair and phosphorylate blunt ends. This was achieved using a combination of enzymes including the NEBNext End Repair Enzyme Mix (New England Biolabs, Ipswich, MA, USA). Following end repair, the DNA fragments underwent 3’ adenylation utilizing the NEBNext dA-Tailing Reaction Mix (New England Biolabs). The adenylated fragments were then ligated with custom adapters containing 5’-methylcytosine instead of 5’-cytosine, along with unique index sequences. The ligation process was performed using the NEBNext Quick Ligation Module (New England Biolabs). Library quantification was carried out using the Qubit 4 Fluorometer and the Qubit dsDNA HS Assay Kit (Thermo Fisher Scientific, Waltham, MA, USA) to ensure accurate DNA concentration measurements. The constructed libraries were then sent to Novogene Corporation, China, for targeted bisulfite sequencing. Upon completion of sequencing, the methylation data was processed and normalized, leading to the generation of beta values for subsequent analysis.

### Receiver operating characteristic (ROC)

A ROC curve was constructed to assess the diagnostic utility, specifically, and sensitivity of the OAS family genes in breast cancer. The analysis was performed using GraphPad Prism (version 9.5.2) software.

### Validation of OAS family genes expression using patients’ data from the Cancer Genome Atlas datasets

UALCAN (http://ualcan.path.uab.edu) and GEPIA (http://gepia.cancer-pku.cn) are invaluable bioinformatics resources for cancer research. UALCAN provides a user-friendly platform for in-depth exploration of cancer transcriptome data, enabling researchers to analyze gene expression, clinical outcomes, and other molecular features across various cancer types [33]. On the other hand, GEPIA facilitates interactive and customizable analyses of gene expression patterns, survival data, and correlation analyses based on The Cancer Genome Atlas (TCGA) and Genotype-Tissue Expression (GTEx) datasets [34]. In the present study, both these tools were used (accessed on July 18, 2024) for the mRNA expression validation of OAS family genes in breast cancer TCGA datasets.

The Human Protein Atlas (HPA) (https://www.proteinatlas.org) is an extensive resource providing comprehensive information on human proteins [35]. Offering detailed tissue and cell-specific expression profiles, immunohistochemistry images, and subcellular localization data, HPA facilitates a deeper understanding of protein expression patterns in normal and disease states, contributing to advancements in biomedical research. In the current study, HPA was used (accessed on July 18, 2024) to validate the protein expression of OAS family genes in breast cancer tissue.

### Promoter methylation validation of OAS family genes using patients’ data from TCGA datasets

OncoDB (http://oncodb.org) is a valuable database for exploring gene expression patterns and methylation data. It allows users to visualize relationships between gene expression, DNA methylation, and clinical outcomes, providing essential insights for cancer research and personalized medicine [36]. In the current research, OncoDB was used (accessed on July 23, 2024) to validate promoter methylation levels of OAS family genes in the TCGA breast cancer dataset.

### Mutational analysis of OAS family genes

cBioPortal (https://www.cbioportal.org) is a pivotal bioinformatics platform for cancer genomics [37]. Offering a user-friendly interface, it enables researchers to explore and visualize complex cancer genomics datasets, fostering insights into genetic alterations, pathways, and clinical implications. The platform provides various analytical tools for examining gene expression, mutations, copy number variations, and epigenetic changes across different cancer types. Researchers can also integrate clinical data with molecular profiles, allowing for a deeper understanding of how genetic alterations influence cancer progression, prognosis, and treatment responses. Additionally, the intuitive visualization features, such as heatmaps, survival plots, and pathway enrichment analysis, help researchers to easily interpret and present their findings. In this work, the cBioPortal database was utilized (accessed on July 26, 2024) for the mutational analysis of OAS family genes in the TCGA breast cancer dataset.

### Prognostic model development

KM plotter (https://kmplot.com) is a vital tool for cancer genomics, simplifying the exploration of complex datasets [38]. Its user-friendly interface enables researchers to uncover genetic alterations and pathway insights, advancing cancer research. In our work, we used this resource (accessed on July 27, 2024) for the survival analysis of OAS family genes across breast cancer patients.

To construct the prediction model, the least absolute shrinkage and selection operator (Lasso) regression analysis was employed using the “glmnet” package in R (https://cran.r-project.org/web/packages/glmnet/index.html). This analysis utilized the TCGA-BRCA dataset as the training set and the GSE97341 dataset as the validation set. Positive coefficients derived from this analysis indicate an increased risk of an event (e.g., death), while negative coefficients indicate a reduced risk. The magnitude of these coefficients reflects the impact of variables on hazard rates, which aids in building prognostic models for survival outcomes.

The prognostic model for breast cancer patient outcomes was defined by the following formula:

$$\:Risk\:score=\sum\:\left(\begin{aligned}&Cox\:regression\:coefficient\cr&\quad\times\:expression\:level\:of\:each\:mRNA\end{aligned}\right)$$

This model provides a risk score by summing the products of the Cox regression coefficients and the expression levels of each mRNA, offering a quantitative measure of prognosis for breast cancer patients.

### OAS family gene expression in diverse immune and molecular subtypes of BRCA

To evaluate the expression of OAS family genes within diverse immune and molecular subtypes of BRCA, we employed (accessed on July 30, 2024) the TISIDB database (http://cis.hku.hk/TISIDB) [39]. TISIDB is an integrated platform that provides comprehensive data on gene expression, immune cell infiltration, and the interactions between genes and immune-related features across multiple cancer types. The database allows researchers to explore the relationship between tumor immunity, gene expression, and clinical outcomes, making it a valuable resource for cancer immunology studies.

### Protein-protein interaction (PPI) network construction and gene enrichment analysis

GeneMANIA (https://genemania.org/) and STRING (https://string-db.org/) are bioinformatics tools used to predict protein-protein interactions (PPI) and gene functions [40, 41]. In this study, both databases were utilized (accessed on November 18, 2024) to construct the PPI network of OAS family genes. Moreover, Venn diagram analysis was conducted to identify shared genes between constructed PPIs. DAVID (https://david.ncifcrf.gov) is a powerful bioinformatics resource for functional genomics [42]. It provides a suite of tools for gene functional annotation, enrichment analysis, and functional annotation clustering, which aids in the comprehensive biological interpretation of large-scale omics data, including transcriptomics, proteomics, and metabolomics. DAVID helps researchers identify biological themes, molecular pathways, and disease associations related to their genes of interest by integrating multiple functional annotation databases. The platform also offers visualization tools, such as enriched GO terms and pathway networks, facilitating the understanding of the biological roles of gene sets in various contexts, including disease mechanisms, drug discovery, and biomarker identification. In this work, DAVID was used (accessed on July 30, 2024) to perform gene enrichment analysis of OAS family genes.

### Knockdown of OAS genes in breast cancer cell line

siRNAs designed to target OAS1, OAS2, OAS3, and OASL genes were synthesized by the OBiO Company. To achieve silencing of OAS1, OAS2, OAS3, and OASL in HOC1 cells, siRNA transfection was performed using INTERFERin Transfection Reagent. Briefly, HOC1 cells were seeded in 6-well plates and allowed to reach 70–80% confluence before transfection. siRNAs (final concentration of 20 nM) were mixed with the appropriate amount of INTERFERin reagent in Opti-MEM (Thermo Fisher Scientific, Catalog Number: 31985062) and incubated for 20–30 min at room temperature to form siRNA-lipid complexes. These complexes were then added dropwise to the cells in complete culture media.

### RT-qPCR and western blotting

To check the efficacy of gene knockdown, RT-qPCR of the OAS family genes was carried out according to the earlier-mentioned conditions. For the Western blot analysis, total proteins from both transfected and control HOC1 cells were extracted using RIPA lysis buffer (Beyotime, Shanghai, China). The protein concentrations were measured with the bicinchoninic acid (BCA) assay (Solarbio Co., Ltd, Beijing, China). Each sample, containing 40 µg of extracted protein, was subjected to separation by 10% SDS-PAGE. Subsequently, proteins were transferred from the gel to a polyvinylidene fluoride (PVDF) membrane (Millipore, Billerica, MA, USA). The membranes were then blocked with 5% nonfat milk for 2–3 h at room temperature (20–25 °C). Primary antibodies were incubated with the membranes overnight at 4 °C. Following primary antibody incubation, the membranes were washed with TBST and then incubated with horseradish peroxidase-conjugated secondary antibodies for 2 h at room temperature (20–25 °C). The enhanced chemiluminescence (ECL) reagent (Millipore, Billerica, MA, USA) was used to visualize the proteins, and the blots were scanned using the ChemiDoc™ XRS system (Bio-Rad Laboratories, Hercules, CA, USA). The gray values of the protein bands were quantified using Image Lab 2.0 software (Genmall Biotechnology Co., Ltd, Wuhan, China), with β-actin (ZSGB-Bio, China) used as a loading control for normalization. Primary antibodies targeting OAS1, OAS2, and OAS3 were obtained from PeproTech (New Jersey, USA), and the anti-OASL antibody was purchased from Abcam (Cambridge, MA, USA). Secondary antibodies were acquired from Zhongshan Golden Bridge Biotechnology (Beijing, China).

### Cell counting Kit-8 and colony formation assays

In the colony formation assay, cells were distributed in 35-mm dishes at a concentration of 300 cells per dish and incubated with the appropriate culture medium for 14 days. Subsequently, cells were fixed and stained with Giemsa solution, and colonies containing a minimum of 50 cells were counted using a microscope. For the CCK-8 assay, cells were seeded into 96-well plates at a density of 3 × 10^3 cells per well. At specified time points (24, 48, 72, or 96 h), 10 µl of CCK-8 reagent was added to each well, followed by a 1-hour incubation at 37 ºC. Absorbance at 490 nm was measured using an automatic microplate reader (BioTek, Vermont, USA).

### Wound healing assay

Cells were cultured in plates until reaching 90% confluence. Subsequently, wounds were created by gently scratching the cell monolayer with a sterile pipette tip. Afterward, the remaining cells were rinsed twice with serum-free culture media, and the closure of the wounds was monitored. Images were captured at 0 and 24 h using a phase-contrast microscope. The percentage of wound closure was calculated by measuring the width of the wound at both time points and comparing the difference, using ImageJ software (version 1.53f).

### Drug prediction analysis

DrugBank (https://www.drugbank.ca) is a comprehensive bioinformatics database providing information on drugs, their targets, and interactions [43]. It serves as a vital resource for researchers, clinicians, and pharmaceutical professionals, facilitating the exploration of drug properties, mechanisms, and potential therapeutic applications. In the present study, the DrugBank database was used (accessed on August 9, 2024) to predict OAS1, OAS2, OAS3, and OASL inhibitory drugs.

### Correlations of OAS family genes with diverse states of breast cancer

The CancerSEA database (http://biocc.hrbmu.edu.cn/CancerSEA) is a comprehensive resource for exploring the functional states of cancer cells at the single-cell level [44]. It integrates data on 14 distinct cancer cell functional states, such as proliferation, apoptosis, and metastasis, across various cancer types. By providing detailed correlations between gene expression and these states, CancerSEA facilitates a deeper understanding of cancer heterogeneity and aids in identifying potential therapeutic targets and biomarkers. In the current study, this database was utilized (accessed on August 9, 2024) to explore correlations of OAS family genes with diverse states of breast cancer.

### Immunolytic and drug sensitivity analysis

The GSCA database (http://bioinfo.life.hust.edu.cn/GSCA) is a robust platform designed for the comprehensive analysis of gene sets in cancer [45]. It integrates multidimensional cancer genomics data to assess gene set expression, mutation patterns, and their clinical relevance across various cancer types. GSCA provides tools for visualizing and interpreting complex data, enabling researchers to identify key pathways and potential therapeutic targets, thereby advancing personalized cancer treatment strategies. In this study, GSCA (accessed on August 10, 2024) was utilized for the immunolytic and drug sensitivity analysis of the OAS genes in breast cancer.

### Statistics

In this study, Gene Ontology (GO) and Kyoto Encyclopedia of Genes and Genomes (KEGG) enrichment analyses were performed using Fisher’s Exact test to identify significant differences [46]. Correlational analyses were conducted using Pearson’s correlation method. For comparative analyses, the Student’s t-test was utilized to compare the means of two independent groups. The t-test was used to evaluate differences in gene expression, clinical outcomes, and other continuous variables between experimental groups. All statistical analyses were performed in GraphPad Prism (version 9.5.2) software. P-value < 0.05 was considered significant.

## Results

### Elevated expression of OAS family genes in breast cancer cell lines

**Fig. 1:**
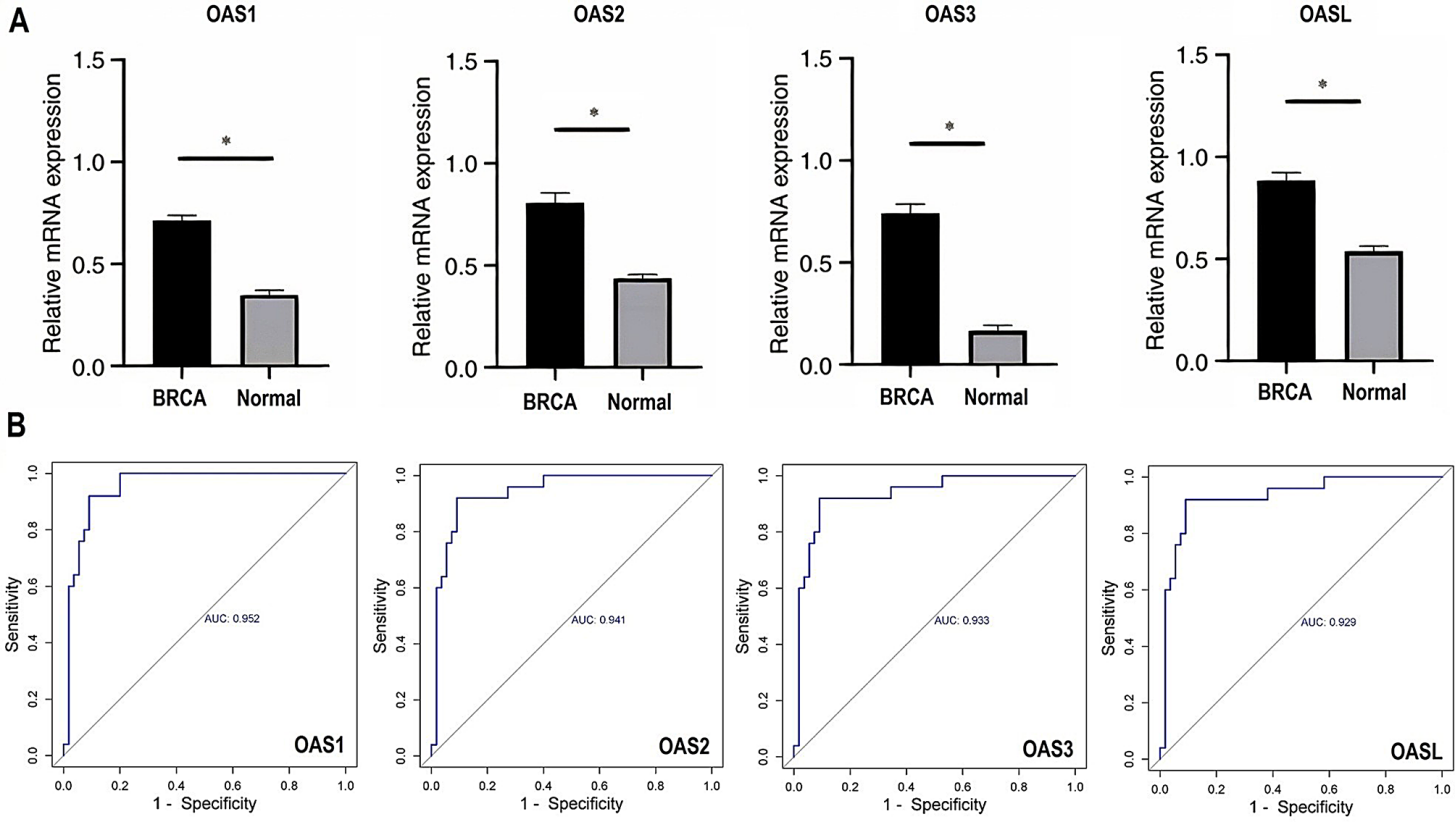
This figure presents the expression analysis of OAS family genes through RT-qPCR and the corresponding Receiver Operating Characteristic (ROC) curves. (**A**) Box plots illustrate the gene expressions of OAS1, OAS2, OAS3, and OASL in both breast cancer and control cell lines. (**B**) ROC curves based on RT-qPCR expression data depict the diagnostic performance of OAS1, OAS2, OAS3, and OASL genes. P*-value < 0.05

We investigated the mRNA expression levels of OAS family genes in breast cancer cell lines (*n* = 14) compared to normal breast cell lines (*n* = 5) using RT-qPCR. The experimental data revealed significantly elevated mRNA levels of OAS1, OAS2, OAS3, and OASL in breast cancer cell lines relative to normal controls (p-value < 0.05, Fig. 1A). These results indicate a potential upregulation of OAS family genes in breast cancer, suggesting their relevance as biomarkers for disease detection.

To further validate the diagnostic potential of the OAS family genes, we performed receiver operating characteristic (ROC) curve analysis based on the RT-qPCR data. The area under the curve (AUC) values for OAS1, OAS2, OAS3, and OASL were determined to be 0.952, 0.941, 0.933, and 0.929, respectively (Fig. 1B). These high AUC values underscore the robustness of these genes in distinguishing breast cancer cell lines from normal breast cell lines with high diagnostic accuracy. The experimental findings, including robust RT-qPCR validation and precise ROC curve analysis, strongly support the role of OAS family genes as reliable transcriptional biomarkers for breast cancer diagnosis.

### Up-regulation of OAS family genes is associated with promoter hypomethylation

**Fig. 2:**
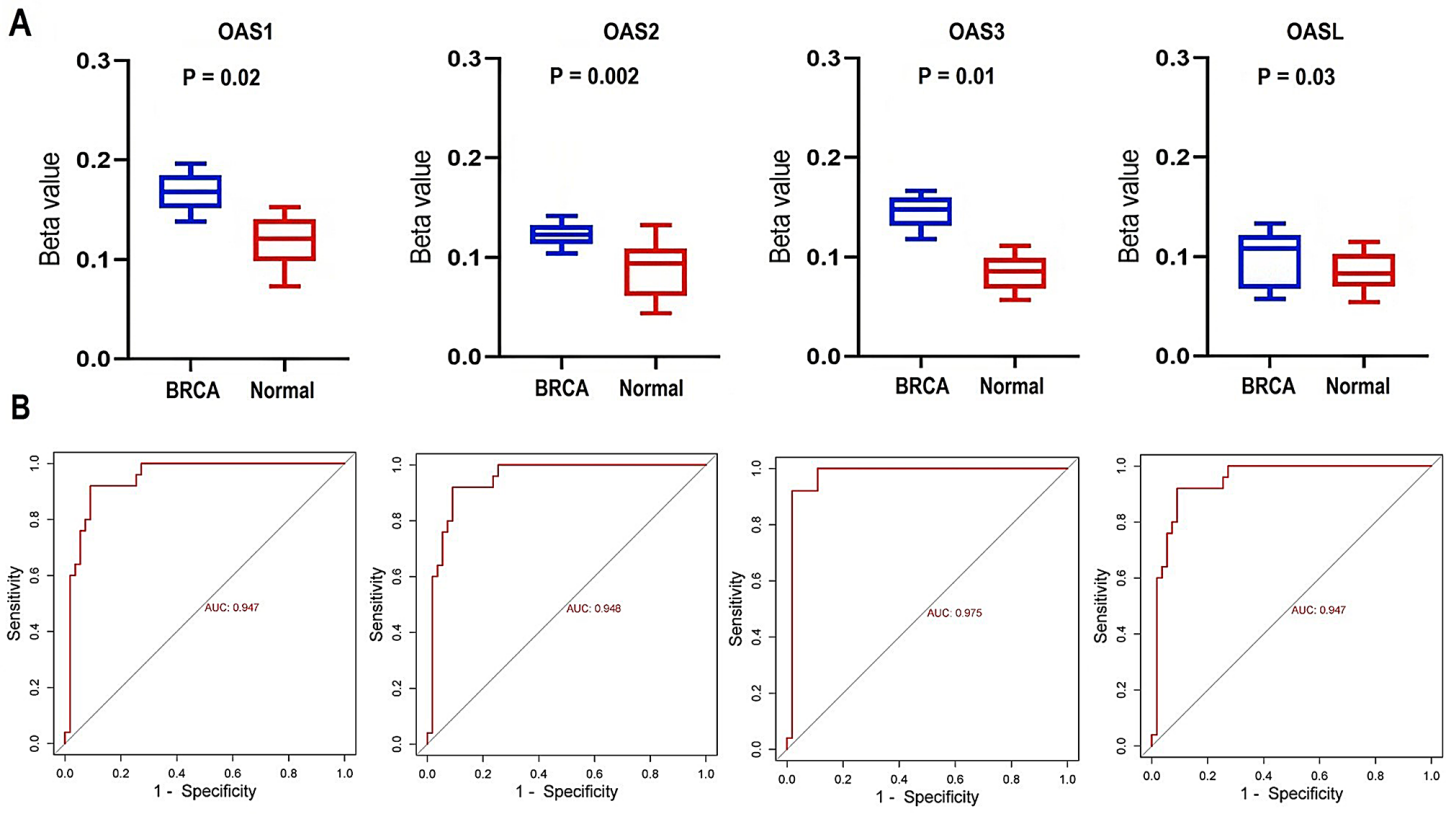
This figure presents the promoter methylation analysis of OAS family genes through bisulfite sequencing and the corresponding Receiver Operating Characteristic (ROC) curves. (**A**) Box plots illustrate the promoter methylation levels of OAS1, OAS2, OAS3, and OASL in both breast cancer and control cell lines. (**B**) ROC curves based on bisulfite sequencing data depict the diagnostic performance of OAS1, OAS2, OAS3, and OASL genes. P-value < 0.05

Aberrant DNA methylation stands out as the most extensively researched epigenetic modification, leading to changes in gene expression and playing pivotal roles in the initiation and progression of carcinogenesis [47, 48]. We hypothesized that the overexpression of OAS family genes in breast cancer might be associated with altered DNA methylation. To experimentally validate this hypothesis, bisulfite sequencing analysis was conducted to assess the promoter methylation levels of OAS1, OAS2, OAS3, and OASL in breast cancer cell lines (*n* = 14) and normal control cell lines (*n* = 5). The analysis demonstrated significantly reduced promoter methylation levels of all OAS family genes in breast cancer cell lines compared to control cell lines (p-value < 0.05, Fig. 2A). These findings suggest a potential epigenetic mechanism underlying the transcriptional upregulation of OAS family genes in breast cancer. To further evaluate the diagnostic relevance of these genes, we performed receiver operating characteristic (ROC) curve analysis based on the bisulfite sequencing data. The calculated area under the curve (AUC) values for OAS1, OAS2, OAS3, and OASL were 0.947, 0.948, 0.975, and 0.947, respectively (Fig. 2B). These high AUC values highlight the diagnostic efficacy of methylation status as a marker for distinguishing breast cancer cell lines from normal cell lines with remarkable accuracy.

The experimental validation, encompassing bisulfite sequencing and ROC curve analysis, provides strong evidence linking reduced promoter methylation to the overexpression of OAS family genes, thereby reinforcing their role as potential epigenetic biomarkers for breast cancer detection.

### Validation of the OAS family genes expression using additional breast cancer cohorts

**Fig. 3:**
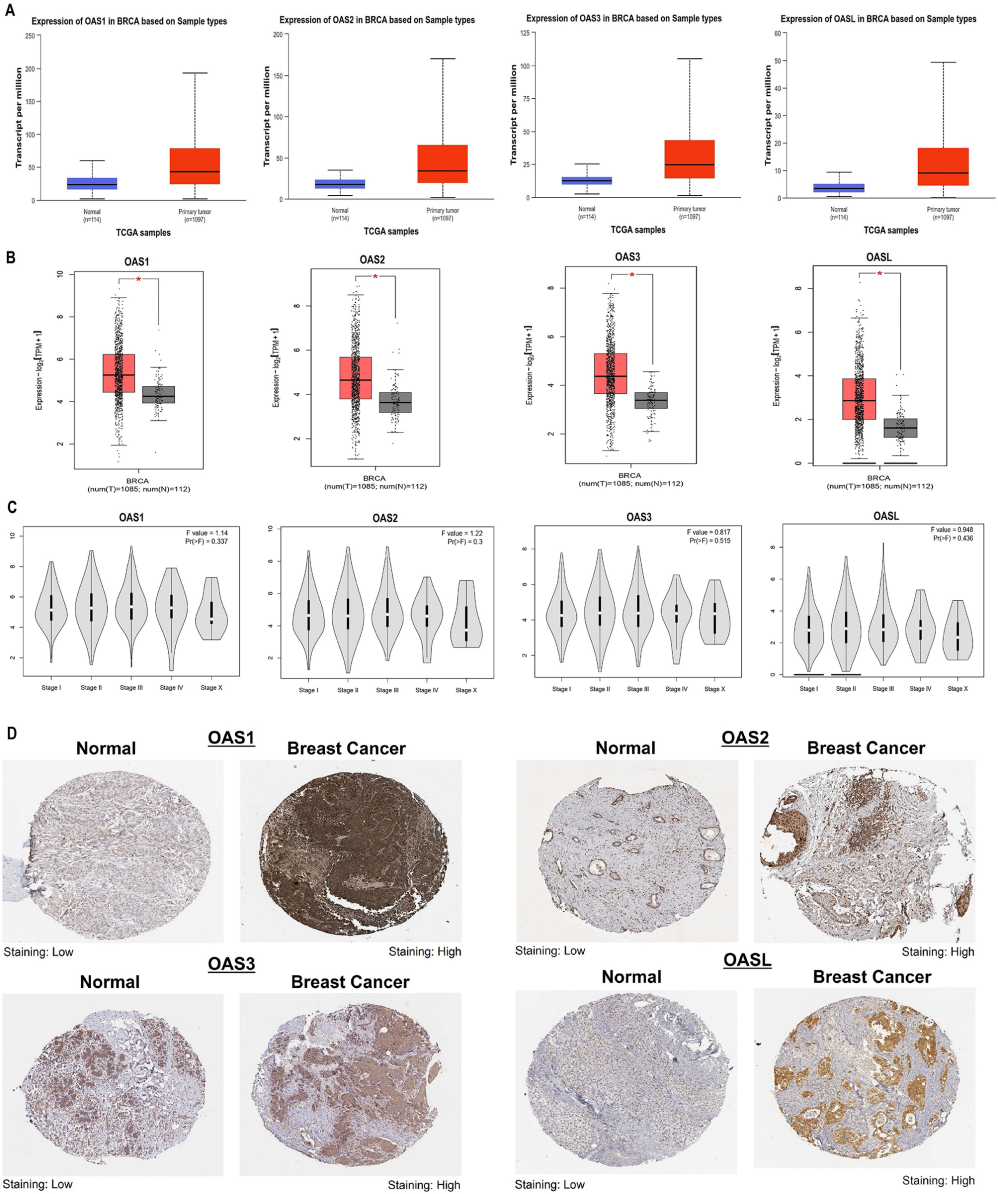
mRNA and protein expression levels validation of OAS family genes in additional The Cancer Genome Atlas cohorts. (**A**) mRNA expression validation of OAS1, OAS2, OAS3, and OASL genes in breast cancer and normal tissue samples via the UALCAN. (**B**) mRNA expression validation of OAS1, OAS2, OAS3, and OASL genes in breast cancer and normal tissue samples via the GEPIA2. (**C)** mRNA expression validation of OAS1, OAS2, OAS3, and OASL genes in breast cancer tissue samples of different stages and normal samples via the GEPIA2. (**D)** Protein expression validation of OAS1, OAS2, OAS3, and OASL genes in breast cancer normal tissues samples via the Human Protein Atlas. P-value < 0.05Please provide high-resolution source file for figure. 3.The Figure 3 with improved resolution is attached as an attachment.

To verify the gene expression findings from the cell lines depicted in Fig. 1, we utilized UALCAN, GEPIA, and HPA databases to examine the expression of OAS1, OAS2, OAS3, and OASL genes at both mRNA and protein levels. Results from UALCAN and GEPIA indicated that the mRNA levels of OAS1, OAS2, OAS3, and OASL were markedly higher (p-value < 0.05) in breast cancer samples compared to normal samples (Fig. 3A-B), aligning with the outcomes observed in cell lines. Furthermore, the findings from GEPIA revealed that the expressions of OAS1, OAS2, OAS3, and OASL genes were notably more pronounced in the advanced stages of breast cancer compared to the early stages (Fig. 3C). Additionally, results from HPA demonstrated a significant elevation in protein expression levels of OAS1, OAS2, OAS3, and OASL in breast cancer tissue samples as opposed to normal tissue samples (Fig. 3D).

### Validation of the OAS family genes promoter methylation levels using additional breast cancer cohort

**Fig. 4:**
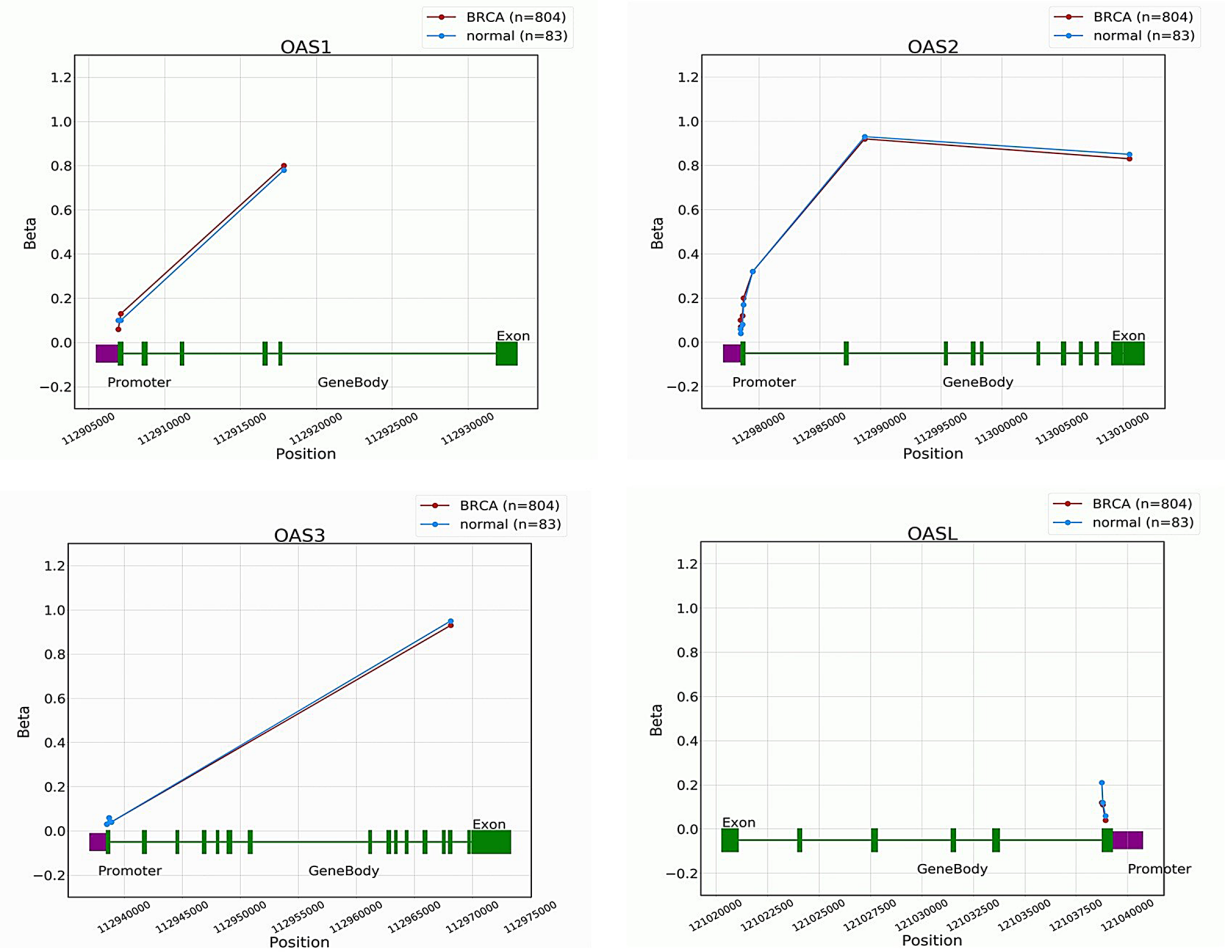
Promoter methylation level validation of OAS family genes in additional The Cancer Genome Atlas breast cancer cohort through the OncoDB database. P-value < 0.05

Next, we validated the promoter methylation levels of OAS1, OAS2, OAS3, and OASL genes in both breast cancer and control samples utilizing the OncoDB database. Our analysis revealed that breast cancer tissues exhibiting elevated gene expression showed a significant decrease in promoter methylation levels (Fig. 4). These results confirmed that the up-regulation of OAS1, OAS2, OAS3, and OASL genes in breast cancer samples is associated with reduced promoter methylation levels.

### Mutational analysis of OAS family genes

**Fig. 5:**
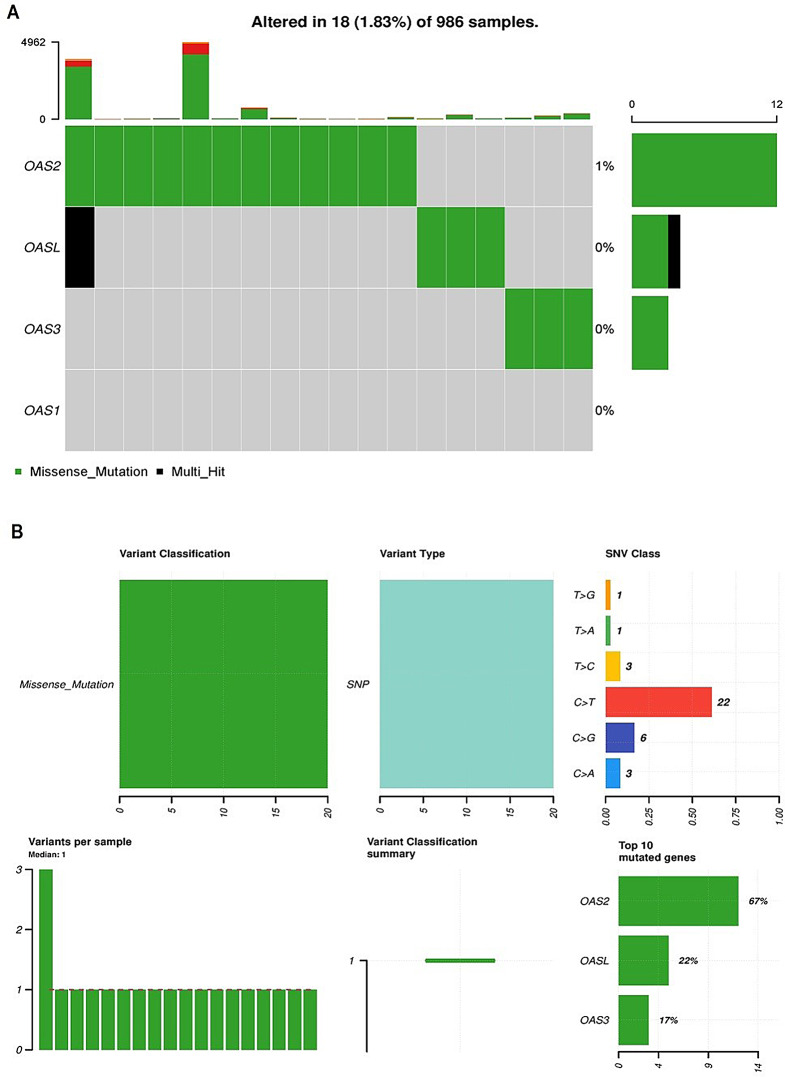
Mutational analysis of OAS family genes in The Cancer Genome Atlas breast cancer cohort via the cBioPortal database. (**A**) Frequency of the observed mutations in OAS genes across breast cancer samples. (**B**) Detailed classification of the observed mutations

The mutational spectrum of OAS1, OAS2, OAS3, and OASL genes in breast cancer patients from the TCGA dataset was investigated using the cBioportal database. The findings indicated that OAS1, OAS3, and OASL genes did not exhibit mutations among the 986 analyzed breast cancer samples (Fig. 5A). In contrast, the OAS2 gene showed mutations in only 1% of the total analyzed 986 breast cancer samples (Fig. 5A). Furthermore, the results revealed that the most prevalent type of mutation observed in the OASL2 gene was C > T mutation (Fig. 5B).

### Prognostic model development

**Fig. 6:**
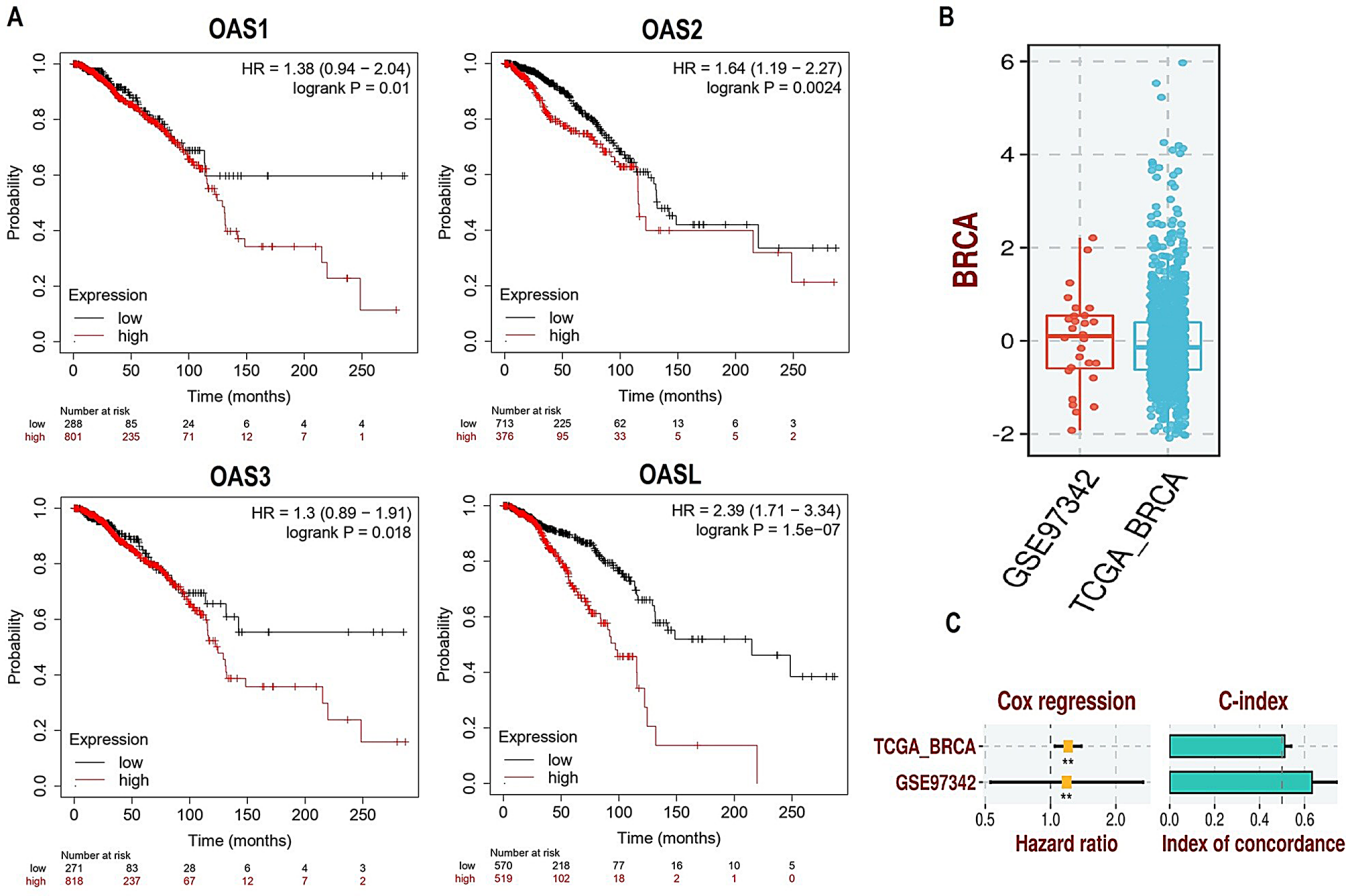
Prognostic analysis and the construction of OAS1, OAS2, OAS3, and OASL genes-based prognostic model. (**A**) KM plotter-based Survival curves of OAS1, OAS2, OAS3, and OASL genes. (**B**-**C**) Risk scores and univariate Cox regression analysis. P-value < 0.05

To investigate the correlation between the expression levels of OAS1, OAS2, OAS3, and OASL genes and the survival outcomes in breast cancer patients, we utilized the Kaplan-Meier Plotter online bioinformatics tool. The results revealed that breast cancer patients exhibiting increased expression of OAS1, OAS2, OAS3, and OASL genes had poor overall survival (Fig. 6A). Furthermore, lasso regression analysis was performed to develop OAS1, OAS2, OAS3, and OASL genes-based predictive model in which the TCGA-BRCA dataset was used as the training set and the GSE97341 dataset was used as the validation dataset. The constructed predictive model showed that OAS1, OAS2, OAS3, and OASL genes can effectively predict the overall survival of breast cancer patients (Fig. 6B-C).

### OAS family gene expression in diverse immune and molecular subtypes of BRCA

**Fig. 7:**
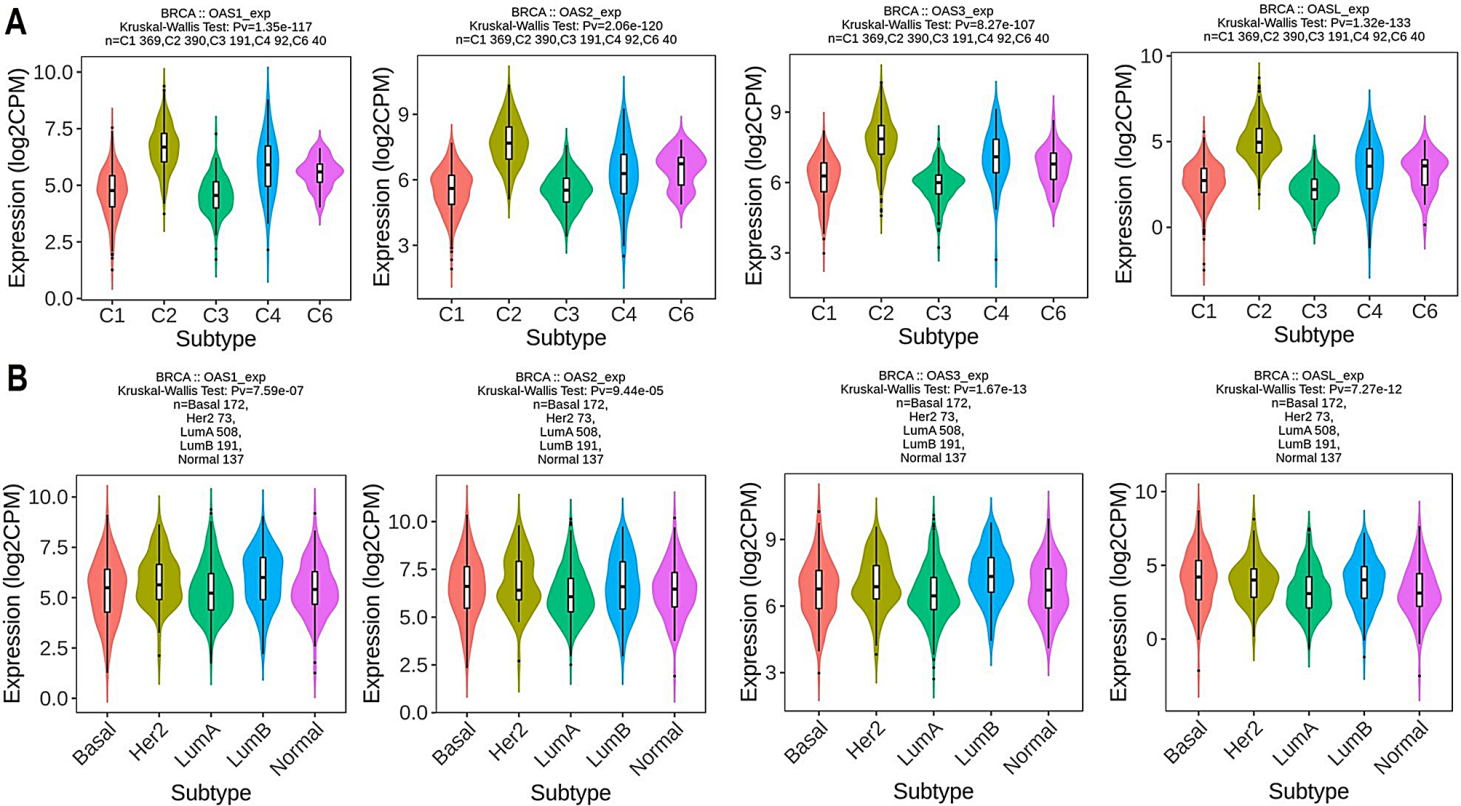
Correlation of OAS family genes expression with diverse immune and molecular subtypes of breast cancer. (**A**) Correlation of OAS1, OAS2, OAS3, and OASL expression with diverse immune subtypes of breast cancer. (**B**) Correlation of OAS1, OAS2, OAS3, and OASL expression with diverse molecular subtypes of breast cancer. P-value < 0.05

Utilizing the TISIDB database, we explored the relationship between OAS1, OAS2, OAS3, and OASL expression in breast cancer across various immune and molecular subtypes. The immune subtypes, denoted as C1 to C6 (representing wound healing, IFN-γ dominant, inflammatory, lymphocyte depleted, immune quiet, and TGF-β dominant), were encompassed in this analysis. The results uncovered a significant correlation between the expression of OAS1, OAS2, OAS3, and OASL and all immune molecule subtypes in breast cancer (Fig. 7A). Additionally, a distinct correlation emerged between hub gene expression and diverse molecular subtypes within breast tumors (Fig. 7B). Taken together, these findings underscore the distinct expression patterns of OAS1, OAS2, OAS3, and OASL in breast tumors across a spectrum of immune and molecular subtypes.

### PPI network construction and gene enrichment analysis

**Fig. 8:**
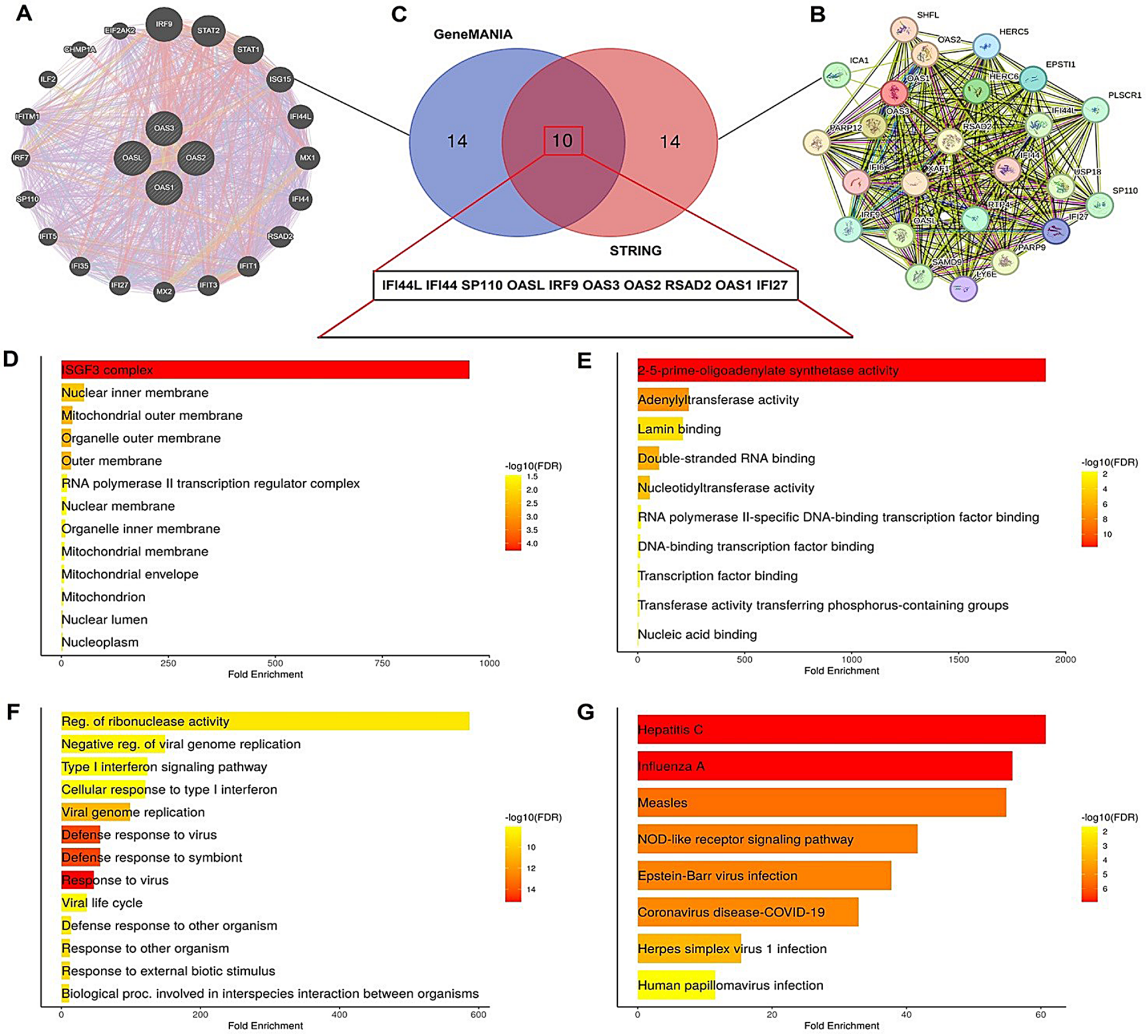
Comprehensive analysis of the protein-protein interaction network and functional enrichment of OAS family genes. (**A**) Protein-protein interaction (PPI) network of OAS family genes and their interactors constructed using the STRING database. (**B**) PPI network of OAS family genes constructed using GeneMANIA. (**C**) Venn diagram showing 10 common genes (IFI44, IFI44L, SP110, OASL, IRF9, OAS3, OAS2, OAS1, RSAD2, and IFI27) identified from STRING and GeneMANIA networks. (**D**) Cellular component enrichment analysis of the common genes. (**E**) Molecular function enrichment analysis of the common genes. (**F**) Biological process enrichment analysis of the common genes. (**G**) Pathway enrichment analysis of the common genes. P-value < 0.05

In this part of our study, we analyzed the PPI and functional roles of OAS family genes. We constructed PPI networks using the STRING (Fig. 8A) and GeneMANIA (Fig. 8B) databases. From these analyses, we identified 10 common binding partners (IFI44, IFI44L, SP110, OASL, IRF9, OAS3, OAS2, OAS1, RSAD2, and IFI27), as shown in the Venn diagram (Fig. 8C). To further investigate the functional roles of these common binding partners, we performed gene enrichment analysis using the DAVID tool. The results revealed significant insights into cellular localization, molecular functions, and biological processes. The cellular component analysis (Fig. 8D) indicated that these proteins are associated with key structures such as the nuclear inner membrane, mitochondrial membrane, and the SFG3 complex, suggesting their involvement in nuclear and mitochondrial functions. Molecular function analysis (Fig. 8E) highlighted activities like 2–5 prime-oligoadenylate synthetase, nucleic acid binding, and transcription factor binding, which are critical in antiviral defense mechanisms. Biological process enrichment (Fig. 8F) revealed that these proteins play a role in processes such as viral genome replication, type I interferon signaling, and defense responses to viral infections. Additionally, pathway analysis (Fig. 8G) showed significant enrichment in viral infection-related pathways, including Hepatitis C, Influenza A, Epstein-Barr virus, and COVID-19, emphasizing their importance in pathogen-host interactions.

### Drug prediction analysis

In this section, we utilized the DrugBank database to anticipate drugs with expression-regulatory potential for the OAS1, OAS2, OAS3, and OASL genes. A total of five drugs, Acetaminophen, Estradiol, Cyclosporine, Calcitriol, and Seocalcitol (Table 1), demonstrated the capability to potentially reduce the expression levels of OAS1, OAS2, OAS3, and OASL, suggesting their prospective use in breast cancer treatment.

**Table 1:** OAS1, OAS2, OAS3, and OASL genes drugs from the DrugBank database

Sr. No	Hub gene	Drug name	Effect	Reference	Group
1	OAS1	Acetaminophen	Decrease expression of OAS1 mRNA	A20420	Approved
2	OAS2	Estradiol	Decrease expression of OAS2 mRNA	A21093	Approved
		Acetaminophen		A20420	
3	OAS3	Cyclosporine	Decrease expression of OAS3 mRNA	A21092	Approved
4	OASL	Calcitriol	Decrease expression of OASL mRNA	A22306	Approved
		Seocalcitol		A22306	

### Correlation of OAS family genes with 14 diverse functional states of breast cancer

**Fig. 9:**
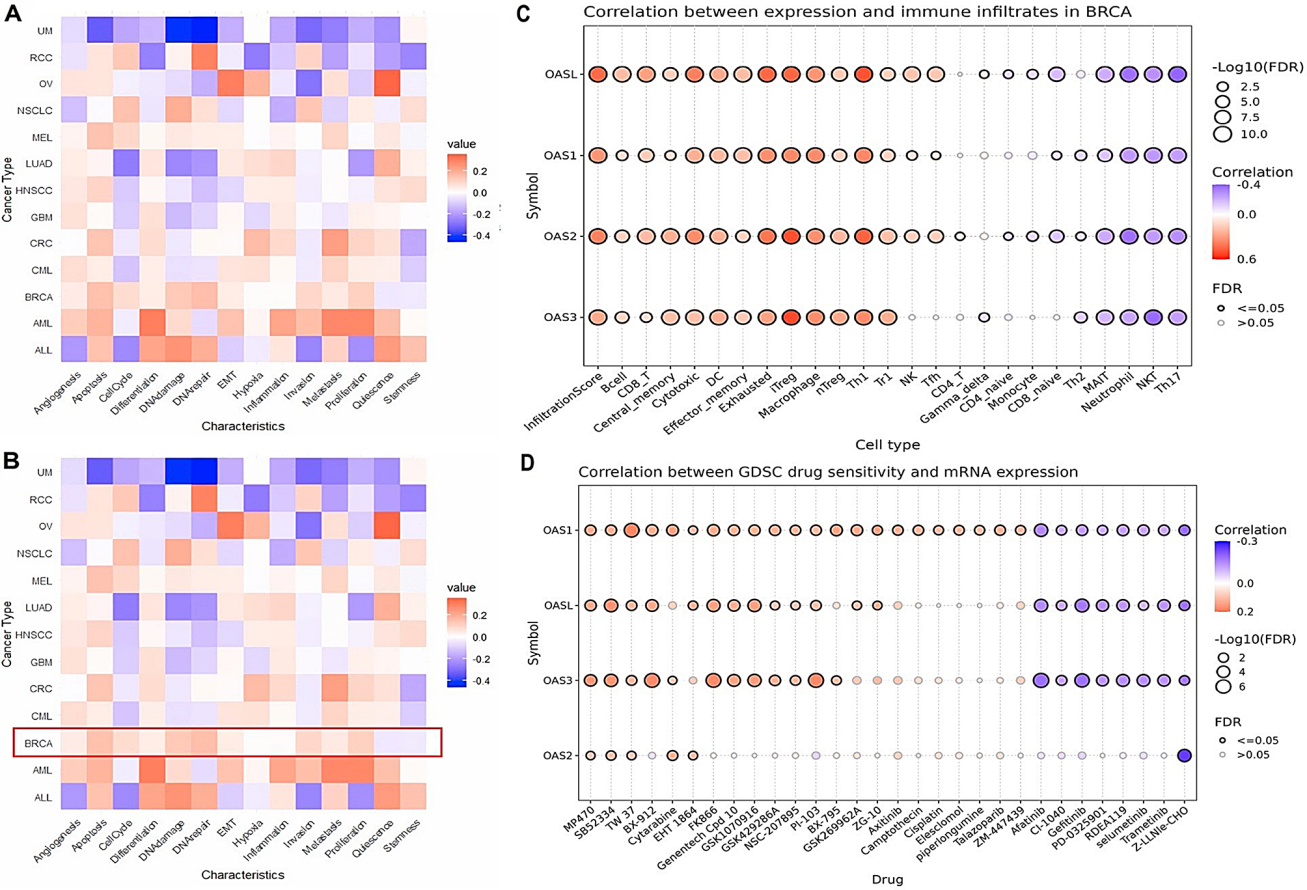
Correlation of OAS family genes with functional states, immunolytic, and drug sensitivity analyses. (**A**-**B**) Correlation of OAS family genes with 14 functional states of breast cancer. (**C**) Correlation of OAS family genes with immune cells in breast cancer. (**D**) Drug sensitivity analysis of OAS family genes. P-value < 0.05

Next, using the CancerSEA database, correlations of OAS family genes were analyzed with 14 diverse functional states of the breast cancer. Figure 9A-B presents a heatmap showing these correlations across multiple cancers. In breast cancer, the OAS genes exhibit a strong positive correlation with inflammation and moderate positive correlations with invasion, metastasis, and stemness (Fig. 9A-B). They also show moderate negative correlations with epithelial-mesenchymal transition (EMT), proliferation, and quiescence (Fig. 9A-B).

### Immunolytic and drug sensitivity analysis of OAS family genes

In this part of our study, firstly we performed immunolytic analysis of OAS family genes in BRCA samples using GSCA database. Results of the analysis showed that OAS family genes in breast cancer are strongly correlated with immune cell infiltrates, such as CD8 T cells and macrophages, underscoring their role in the immune response within the tumor microenvironment (Fig. 9C). Lastly, Fig. 9D shows the correlation between OAS gene expression and drug sensitivity from the GDSC dataset, revealing that these genes are strongly linked to various chemotherapeutic agents. This suggests that OAS family genes could serve as biomarkers for predicting drug response in breast cancer, aiding in the development of personalized treatment strategies.

### Down-regulation of OAS1, OAS2, OAS3, and OASL inhibit HOC1 breast cancer cell growth

**Fig. 10:**
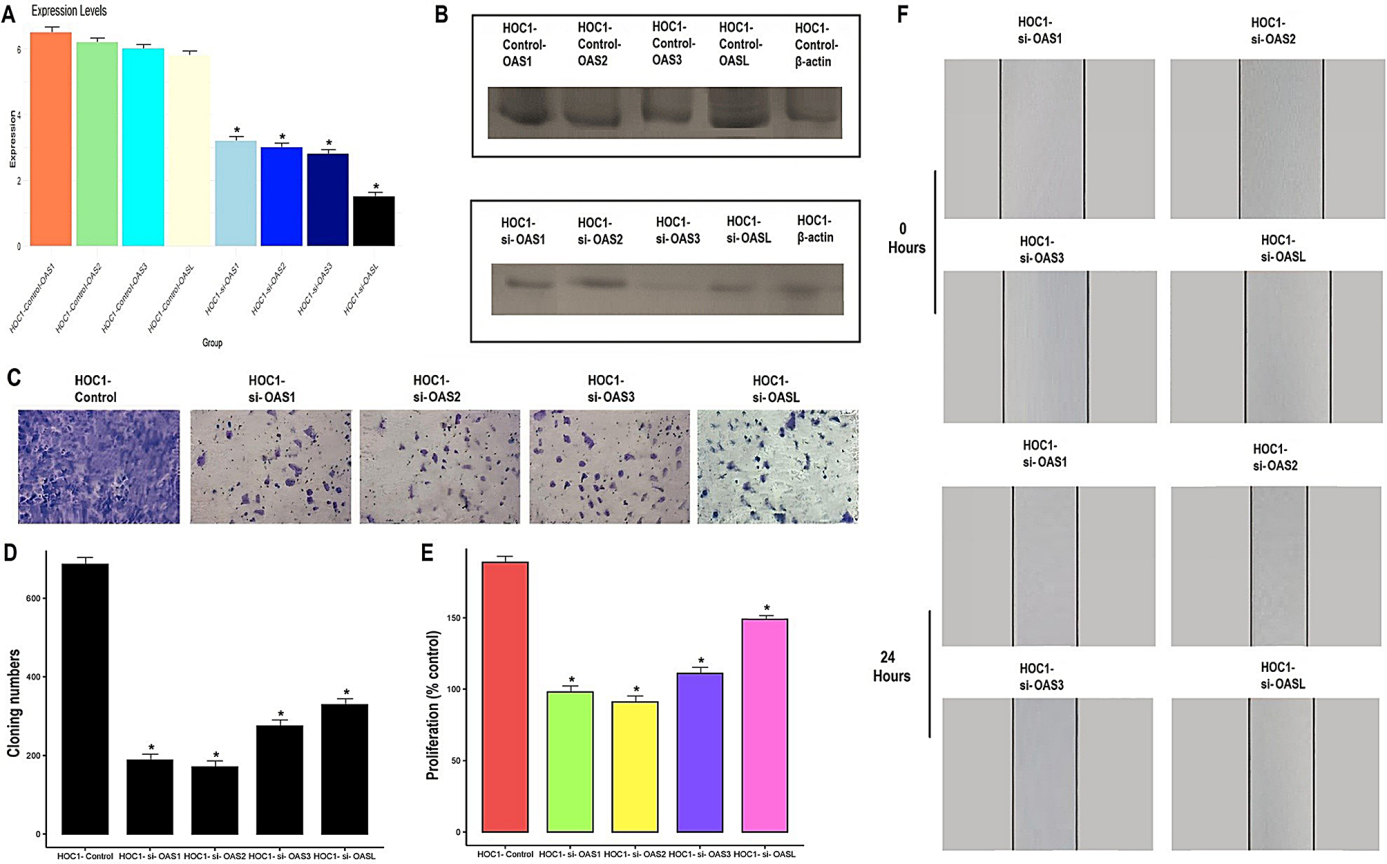
Knockdown of OAS1, OAS2, OAS3, and OASL impairs the growth and metastatic potential, and enhance wound healing ability of the HOC1 Cells. (**A**-**B**) The transfection efficiency of OAS1, OAS2, OAS3, and OASL was checked with the help of RT-qPCR and Western blot analyses. (**C**) HOC1 control and transfected cells were analyzed for colony formation, (**D**-**F**) Proliferation and wound healing. P*-value < 0.05

To further investigate the functional roles of the OAS family genes in breast cancer, we designed four siRNAs targeting OAS1, OAS2, OAS3, and OASL and transfected them into HOC1 cells. The knockdown efficiency of each siRNA was validated through qRT-PCR and Western blot analyses, confirming significant downregulation at both mRNA and protein levels (Fig. 10A-B). Functional assays were conducted to assess the impact of silencing these genes on cellular behavior. The colony formation assay revealed a marked reduction in colony numbers following the knockdown of OAS1, OAS2, OAS3, and OASL, indicating their essential role in promoting cell proliferation and survival (Fig. 10C-D). Additionally, CCK-8 assays demonstrated a significant decrease in the proliferation rate of HOC1 cells upon gene silencing (Fig. 10E). Interestingly, the wound healing assay showed that the knockdown of these genes enhanced the migratory capacity of HOC1 cells (Fig. 10F). Overall, these results highlight the critical roles of OAS1, OAS2, OAS3, and OASL in supporting the growth and proliferation of breast cancer cells, while also suggesting their involvement in modulating cellular migration.

## Discussion

Recent studies have suggested a link between the OAS gene family and breast cancer, particularly regarding immune modulation and tumor progression [49–52]. However, further in-depth investigations are needed to confirm and clarify these associations. In our study, we used a combination of bioinformatics tools and molecular experiments to comprehensively examine the involvement of OAS genes in breast cancer. By analyzing gene expression, genetic alterations, and associations with molecular and immune subtypes, we provide new insights into how OAS genes influence breast cancer biology. This research could contribute to identifying potential biomarkers and therapeutic targets for more personalized treatment strategies.

The OAS gene family consists of key enzymes involved in the cellular antiviral response [17]. This family includes OAS1, OAS2, OAS3, and OASL, each encoding proteins that play a role in detecting double-stranded RNA (dsRNA), a common feature of many viruses [53]. Upon recognizing dsRNA, these proteins catalyze the synthesis of 2’-5’ oligoadenylates from ATP. These oligoadenylates then activate RNase L, which degrades viral RNA and inhibits viral replication, thereby playing a critical role in the innate immune response [18]. The dysregulation of OAS genes has been observed in various cancers, reflecting their complex role in tumor biology. In breast cancer, for instance, there is a notable upregulation of OAS genes such as OAS1, OAS2, OAS3, and OASL [30, 54]. This increase in expression is often linked to promoter hypomethylation, indicating that epigenetic modifications can affect gene activity [54]. High levels of OAS gene expression in breast cancer are associated with poorer prognosis, suggesting their potential utility as diagnostic and prognostic biomarkers [29]. Similarly, in lung cancer, alterations in OAS gene expression have been associated with tumor progression and response to therapy [22, 55]. Variations in OAS expression can influence tumor immunity and contribute to the development of resistance to conventional treatments. For example, reduced activity of OAS genes can impair the antiviral defense mechanisms of cells, potentially allowing for increased tumor growth [56]. In prostate cancer, dysregulation of OAS genes involves both upregulation and downregulation, which can significantly impact tumor behavior [57]. Increased expression of OAS genes in prostate cancer is often associated with more aggressive tumor phenotypes, whereas decreased expression can affect the cancer cells’ ability to mount an effective immune response [57]. The present study identified high expression of OAS family genes in breast cancer and its association with some important biological processes of breast cancer. High expression of OAS family genes was associated with the worst survival of breast cancer patients. Furthermore, our findings indicate a noteworthy negative correlation between OAS1, OAS2, OAS3, and OASL elevated gene expression and a substantial decrease in promoter methylation levels across breast cancer tissues. These results reinforce the notion that the up-regulation of OAS1, OAS2, OAS3, and OASL genes in breast cancer is closely associated with a reduction in promoter methylation. This correlation aligns with existing knowledge implicating DNA methylation as a crucial epigenetic mechanism influencing gene expression [58, 59]. Although the up-regulation of OAS family genes in breast cancer has been previously reported, our study offers novel insights by linking this overexpression to promoter hypomethylation, a key epigenetic modification not explored in earlier breast cancer research. Moreover, this study is the first to report the expression patterns of OAS family genes in a diverse panel of breast cancer cell lines.

The comprehensive examination of the mutational landscape of OAS1, OAS2, OAS3, and OASL genes in breast cancer patients revealed that OAS1, OAS3, and OASL exhibited an absence of mutations in all 986 analyzed breast cancer samples. In contrast, the OAS2 gene displayed mutations in a minimal 1% of the total samples, suggesting a relative rarity of genetic alterations in this gene within the breast cancer cohort. These findings emphasize the genetic stability of OAS1, OAS3, and OASL genes in breast cancer, highlighting OAS2 as the primary gene with observed mutations and underscoring the potential significance of specific mutation types in OASL2 within the context of breast cancer genetics. Previous studies have reported that OAS genes, particularly OAS1 and OAS3, are often involved in antiviral responses and immune regulation [17, 60] but have not been extensively studied for their mutational frequency in cancers. In contrast, studies on other cancer types, such as lung and colorectal cancer, have observed OAS gene mutations, particularly in OAS1 and OAS2 [32, 61], though these alterations are relatively rare. Further exploration of these mutations may contribute to a deeper understanding of their functional implications in breast cancer pathogenesis.

Our study observed significant correlations between the expression of OAS1, OAS2, OAS3, and OASL and various molecular and immune subtypes of breast cancer. This finding aligns with previous research that has linked OAS genes to immune responses and tumor progression in different cancer types, including breast cancer [29, 62]. For example, studies have suggested that the OAS family plays a role in modulating the immune response in cancers through their involvement in the interferon pathway and antiviral defense mechanisms [30]. However, while prior studies have focused on the individual roles of OAS genes in immune modulation and tumor biology [30], few have investigated the specific relationship between these genes and breast cancer molecular and immune subtypes [49].

In contrast to earlier studies, which primarily examined gene expression in a broader context or in relation to immune infiltration, our study uniquely correlates the expression of OAS genes with distinct breast cancer subtypes, both molecular and immune, providing a more granular understanding of their potential roles in shaping the breast cancer tumor microenvironment. While some studies have indicated altered expression of OAS genes in breast cancer tissues [51, 63], our study goes further by linking these expression patterns to specific breast cancer subtypes, suggesting a possible subtype-specific role of OAS genes in immune modulation and tumor progression.

Moreover, this study revealed that up-regulated OAS1, OAS2, OAS3, and OASL genes were associated with the dysregulation of diverse signaling pathways, including “Hepatitis C, Influenza A, Epstein-Barr virus, and COVID-19 signaling pathway” etc. The role of these pathways in the development of various cancers is already well studied [64–68]. To the best of our knowledge, this study is the first to explore the association of OAS genes with these pathways in breast cancer.

The knockdown of OAS1, OAS2, OAS3, and OASL in HOC1 cells, validated by qRT-PCR and Western blot analyses, revealed their critical roles in breast cancer. Gene silencing significantly reduced colony formation and proliferation, highlighting their involvement in promoting cell growth and survival. Notably, wound healing assays showed enhanced cellular migration upon knockdown, suggesting these genes may also influence migration dynamics. This finding aligns with previous studies indicating that OAS family members are not only interferon-stimulated genes with antiviral properties but also play a role in cellular growth and transformation in malignancies [69, 70]. These observations are intriguing as it suggests a potential compensatory mechanism or a shift in cellular dynamics upon the loss of OAS family gene function. Such paradoxical effects have been reported in other cancers, where the silencing of tumor-promoting genes unexpectedly enhances migration due to complex feedback loops or altered signaling pathways [71, 72]. These experimental findings collectively highlight the dual roles of OAS family genes in breast cancer progression. On one hand, they are essential for maintaining proliferation and survival; on the other hand, they may influence migration through indirect mechanisms. Further investigation is required to delineate the molecular pathways mediating these effects, including the potential involvement of interferon signaling and downstream effector molecules.

This study highlights the OAS family genes as potential biomarkers for breast cancer diagnosis and prognosis, showcasing their elevated expression and association with promoter hypomethylation. The use of multiple datasets for validation strengthens the reliability of the findings. However, the study’s limitations include its reliance on cell lines and retrospective data, which may not fully capture the complexity of in vivo conditions. Moreover, while the study identifies promising therapeutic targets, further research is needed to evaluate the efficacy of targeting OAS genes in clinical trials to confirm their therapeutic potential and applicability in personalized medicine.

## Conclusion

This study examined the role of the OAS gene family in breast cancer, highlighting a strong association between elevated expression levels and reduced overall survival. The findings offer important insights into the biomarker potential and molecular mechanisms of the OAS gene family, enhancing our understanding of their significance and therapeutic value in breast cancer.

## Data Availability

Publically analyzed datasets in this study can be found at following links: For the validation of OAS family gene expression, UALCAN (http://www.ualcan.path.uab.edu/) and GEPIA (http://www.gepia.cancer-pku.cn/) were used. For protein expression, The Human Protein Atlas (HPA) (www.proteinatlas.org/) was employed. Promoter methylation was validated with OncoDB (http://www.oncodb.org/). Mutational analysis was conducted using cBioPortal (http://www.cbioportal.org/). Survival analysis was performed with KM Plotter (http://www.kmplot.com/analysis/). TISIDB (http://www.tisidb.org/) was utilized for immune and molecular subtype evaluation. Gene enrichment analysis was done using DAVID (http://david.ncifcrf.gov/). Drug prediction was conducted with DrugBank (http://www.drugbank.ca/). Functional state analysis was performed using CancerSEA (http://www.biocc.hrbmu.edu.cn/CancerSEA/), and GSCA (http://www.bioinfo.life.hust.edu.cn/GSCA/) was used for immunolytic and drug sensitivity analysis.
